# Transcriptome Responses of Insect Fat Body Cells to Tissue Culture Environment

**DOI:** 10.1371/journal.pone.0034940

**Published:** 2012-04-06

**Authors:** Norichika Ogata, Takeshi Yokoyama, Kikuo Iwabuchi

**Affiliations:** 1 Laboratory of Applied Entomology, Faculty of Agriculture, Tokyo University of Agriculture and Technology, Tokyo, Japan; 2 Laboratory of Sericultural Science, Faculty of Agriculture, Tokyo University of Agriculture and Technology, Tokyo, Japan; New Mexico State University, United States of America

## Abstract

Tissue culture is performed to maintain isolated portions of multicellular organisms in an artificial milieu that is outside the individual organism and for considerable periods of time; cells derived from cultured explants are, in general, different from cells of the corresponding tissue in a living organism. The changes in cultured tissues that precede and often explain the subsequent cell proliferation of explant-derived cells have been partially studied, but little is known about the molecular and genomic basis of these changes. Comparative transcriptomics of intact and cultured (90 hours in MGM-450 insect medium) *Bombyx mori* tissues revealed that fewer genes represented a larger portion of the transcriptome of intact fat body tissues than of cultured fat body tissues. This analysis also indicated that expression of genes encoding sugar transporters and immune response proteins increased during culture and that expression of genes encoding lipoproteins and cuticle proteins decreased during culture. These results provide support for hypotheses that cultured tissues respond immunologically to surgery, adapt to the medium by accelerating sugar uptake, and terminate their identity as part of an intact organism by becoming independent of that organism.

## Introduction

Tissue culture was devised as a method for studying the behavior of plant and animal cells in an environment that is free from systemic variations that might arise *in vivo* both during normal homeostasis and under the stress of an experiment. Today, this technique is essential for cell engineering. More than 500 insect cell culture lines have been established and continuously maintained [1]; these lines are used as research tools in virology, immunology, and physiology, and several cell lines are used commercially to produce recombinant proteins of biomedical significance. Since most insect cells have tolerance against changes in temperature [2], pH [3], and osmotic pressure [4], many of these cell lines are very useful as cell culture models of cellular phenomena. However, no general method has been developed to establish a cell line from an arbitrary tissue of an arbitrary insect species. Cell lines are established from primary culture of tissue when a population of proliferating cells derived from the primary tissue explant undergo immortalization [5]. In primary cultures of insect cells, it usually takes several months for active cell proliferation to start [6]. It is thought that during the early stage of primary culture, isolated explants activate immune responses to the culture conditions and then, ultimately, some subpopulation of cells adapts to the culture conditions. In mammalian tissue culture, the changes in the explant itself are the subject of considerable discussion [7]. Hadda (1912) noted that the cells derived from the explant are different from cells in the primary tissue. Champy (1913) proposed that two the phenomena, cell proliferation and cell dedifferentiation, are essential to establishment of tissue cultures. While many tissues in adult animals rarely show evidence of mitosis, active cell division does occur in culture, and this enhanced cell division is accompanied by dedifferentiation. However, the lack or loss of primary tissue characteristics in cultured cells is not primarily due to dedifferentiation, but rather to selective overgrowth of specific cells [8]. Dedifferentiation and selective cell survival and proliferation are clearly two important changes that take place as subpopulation of cells adapt to tissue culture. Recently it is widely known that there are many differences in cell behavior between cultured cells and their counterparts *in vivo* [9], but many of the changes that occur during this adaptation remain unclear. With the introduction of next-generation sequencing [10], genomic sequence data from many species became available, and profiling of whole transcriptomes became possible [11]. This type of profiling could provide new information on the reaction of insect cell as they respond to artificial culture conditions. The silkworm, *Bombyx mori*, is the first Lepidoptera for which a genome sequence has been openly released [12]. The *B. mori* genome is 368 Mb, and there are 14,623 predicted genes in the current version of the annotated sequence. Although annotation has just started and not all the genes are correctly predicted, this species was useful for transcriptome analysis. Fat body is principally responsible for intermediary metabolism and nutrient storage in insects. Here, the transcriptomes of intact fat body tissues and cultured fat body tissues were compared to evaluate the effects of culturing on genome-wide transcription in an insect tissue.

## Results

**Figure 1:**
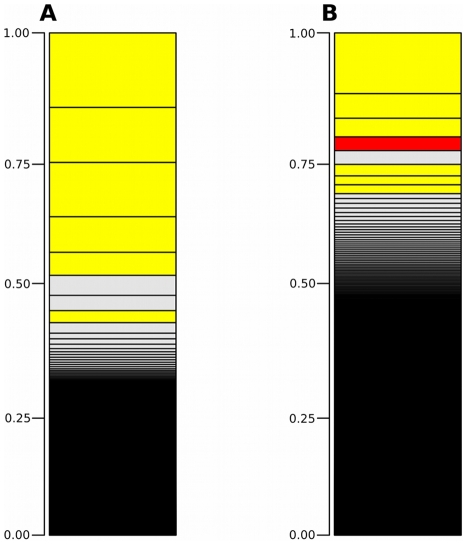
Bar charts of transcriptome of silkworm fat body. Bar charts of transcriptome based on RPKM. Yellow: Lipoproteins BGIGMGA004399, BGIGMGA004394, BGIGMGA004395, BGIGMGA004396, BGIGMGA004397 (in no particular order). Red: Heat shock protein like-protein BGIGMGA000578. White: other genes. Over 14,000 genes are in these bar chart, most genes are invisible and included in black region. A: Transcriptome of intact fat body, B: Transcriptome of cultured fat body.

**Figure 2:**
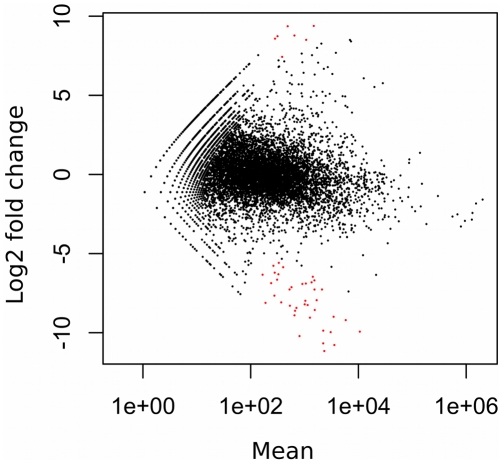
Differential expression analysis of intact fat body and cultured fat body. Scatter plot of log2-fold changes versus mean. Genes marked in red were detected as differentially expressed at the 5% false-discovery rate (FDR) using DESeq. A = log2((Cultured/Nc ⋅ Intact /Ni)^1/2^), M = log2(Cultured/Nc)−log2(Intact/Ni).

**Figure 3:**
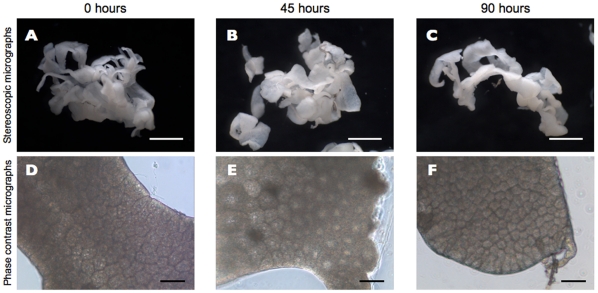
Stereoscopic micrographs and phase contrast micrographs of silkworm fat body tissue during culture. (A-C) Stereoscopic micrographs of silkworm fat body tissue during culture. Scale bars are 1 mm. (A) Fat body tissue immediately after dissection. (B) Fat body tissue 45 hours after culture. (C) Fat body tissue 90 hours after culture. (D–F) Phase contrast micrographs of silkworm fat body tissue during culture. (D) Fat body cells immediately after dissection. (E) Fat body cells 45 hours after culture. (F) Fat body cells 90 hours after culture.

Samples of total RNA were prepared from acutely dissected intact silkworm larval fat body and from fat body tissues that had been cultured for 90 hours in MGM-450 insect medium. Each sample was sequenced on a separate single lane of a flow cell. Sequencing resulted in 23-29 million 36-base-pair reads per lane (Table 1) passing Illumina’s quality filter; in all, 53 million reads and 7.9 GB of silkworm fat body transcript sequence data were generated. Mapping analysis was performed using CASAVA 1. 7. 0 [13]; 17 095 689 (58%) intact fat body and 14 257 793 (60%) cultured fat body high quality reads were mapped to GLEAN loci in the silkworm genome. Based on the KAIKObase [14] annotation, there are 14,623 predicted genes in the annotated silkworm genome; 9,850 genes of these were expressed in intact fat body, and 11,106 genes in cultured fat body. Expression of 11,451 genes was detected, and 9,505 of these genes were expressed in both samples. Reads per kilobase of exon model per million mapped reads [11] (RPKM) were calculated for all genes using CASAVA 1. 7. 0. In intact fat body, lipoprotein-coding genes (BGIBMGA004399, BGIBMGA004394, BGIBMGA004396, BGIBMGA004457, BGIBMGA004397, BGIBMGA004395) occupied half of the transcriptome (Fig. 1A). In cultured fat body, these genes occupied about a quarter of the transcriptome (Fig. 1B). The expression of a gene encoding a heat shock protein-like protein (BGIBMGA005781) occupied a larger portion of the cultured fat body transcriptome than of the intact fat body transcriptome. Fewer genes represented a lager portion of the transcriptome of intact fat body than of cultured fat body (Fig. 1A and 1B). To compare heterogeneity of both transcriptomes, Shannon’s entropy [15] was calculated for all RPKMs. Shannon’s entropy was higher in the sample from cultured fat body (6.488197 shannon in intact fat body and 8.518913 shannon in cultured fat body) indicating that heterogeneity decreased during culture. The relative RNA production in the two tissue samples was estimated using edgeR [16] which is an R software package for statistical analysis [17], and based on this analysis, total RNA production in fat body was reduced by two-fifths during culture. We found 54 differentially expressed genes (false discovery rate (FDR) < 0.05) with DESeq [18] using read-count data (Fig. 2). During culture, expression of 11 genes increased (Table 2), and expression of 43 genes decreased (Table 3). The morphology of cultured fat body tissues were examined by stereoscopic microscope and phase contrast microscope (Fig. 3). There was no crucial difference.

**Table 1:** Read-mapping statistics for 2 lanes sequenced on one flow cell.

Sample	Reads	Mapped Reads	% Aligned
Intact fat body	29,532,006	17,095,689	57.89%
Cultured fat body	23,938,758	14,257,793	59.56%

**Table 2:** Differentially expressed *Bombyx mori* genes for which expression increased during culture of fat body.

Gene ID	Intact	Cultured	Log Ratio	FDR	Seq. Description	eValue	ACC
BGIBMGA004427	0	865	Inf	0.007777171	sugar transporter-like protein	4.65E-07	XP_001850640
BGIBMGA004869	0	736	Inf	0.010526917	protease inhibitor-like protein	2.03E-22	ABG72724
BGIBMGA004426	1	1431	9.36439731	0.012002245	sugar transporter-like protein	4.91E-06	XP_001658148
BGIBMGA001007	1	925	8.734898908	0.020018747	monocarboxylate transporter-like protein	1.60E-86	XP_001659154
BGIBMGA012864	2	1907	8.778678481	0.020177428	peptidoglycan recognition protein-like protein	4.00E-16	BAF03520.1
BGIBMGA001347	1	832	8.582029071	0.022363186	fibroin p25-like protein	1.06E-125	BAB39500
BGIBMGA002314	0	497	Inf	0.027138743	farnesoic acid o-methyl transferase-like protein	1.24E-26	ABF18352
BGIBMGA001324	0	490	Inf	0.027857698	juvenile hormone binding protein e96h-0303	1.67E-136	NP_001036949.1
BGIBMGA007462	3	4360	9.386739272	0.039513702	sodium-dependent phosphate transporter-like protein	0	XP_001662636.1
BGIBMGA001358	3	1124	7.431053173	0.04523946	–NA–	-	-
BGIBMGA013866	4	3152	8.503641173	0.046425762	gloverin-like protein	3.25E-18	NP_001037684.1

“Intact" and “Cultured" columns indicate the number of read counts in intact fat body and cultured fat body of *Bombyx mori*.

**Table 3:** Differentially expressed *Bombyx mori* genes for which expression decreased during culture of fat body.

Gene ID	Intact	Cultured	Log Ratio	FDR	Seq. Description	eValue	ACC
BGIBMGA001030	1093	2	-10.21248833	0.002673821	troponin i	1.49E-31	ACN86369.1
BGIBMGA000329	3010	4	-10.67395842	0.003888079	larval cuticle protein LCP-30 precursor	1.33E-99	NP_001037490.1
BGIBMGA002549	881	4	-8.901408856	0.003888079	cuticular protein RR-1 motif 5	2.84E-47	NP_001166743.1
BGIBMGA009184	902	5	-8.613466175	0.003888079	cathepsin-like protein	1.69E-58	NP_001164088.1
BGIBMGA014226	3156	3	-11.15732964	0.003888079	myosin heavy chain-like protein	0	EFN88457.1
BGIBMGA010112	179	0	-Inf	0.004045802	–NA–	-	-
BGIBMGA011714	499	4	-8.081306652	0.004045802	cuticular protein hypothetical 27	3.00E-25	NP_001166750
BGIBMGA008023	723	5	-8.294334389	0.004190128	–NA–	-	-
BGIBMGA000333	3013	7	-9.868040683	0.004206699	larval cuticle protein LCP-22 precursor	7.48E-84	NP_001036828.1
BGIBMGA000613	1706	7	-9.047457656	0.004206699	miniparamyosin	0	ACM17460.1
BGIBMGA008255	956	6	-8.434314954	0.004206699	cutilcle protein-like protein	1.00E-132	NP_001036893
BGIBMGA011766	4849	6	-10.77691968	0.004501388	cuticular protein hypothetical 25	7.00E-119	NP_001166752.1
BGIBMGA000341	372	4	-7.657569458	0.005459271	cuticular protein RR-1 motif 28	3.39E-35	NP_001166722.1
BGIBMGA001031	255	2	-8.112764084	0.005537234	troponin i	4.07E-50	ACN86368.1
BGIBMGA005278	1361	10	-8.206933904	0.00583966	cuticular protein RR-1 motif 3-like protein	5.29E-40	NP_001166744.1
BGIBMGA000612	1544	11	-8.251436066	0.006132684	paramyosin	0	ACF21977.1
BGIBMGA011765	4138	9	-9.963203575	0.006132684	cuticular protein hypothetical 24	3.00E-70	NP_001166753
BGIBMGA001587	2093	12	-8.564804743	0.006659562	tropomyosin 1	5.07E-137	NP_001040445.1
BGIBMGA006510	1397	12	-7.981564452	0.006659562	myosin light chain-like protein	2.38E-60	AAV91411.1
BGIBMGA006937	710	10	-7.268157767	0.007777171	troponin c	7.06E-58	NP_001037594.1
BGIBMGA004065	120	0	-Inf	0.010456244	chemosensory protein 5	2.46E-60	NP_001037062.1
BGIBMGA004893	771	12	-7.124035196	0.010526917	fau, isoform B-like protein	7.30E-18	NP_731506.1
BGIBMGA010235	323	6	-6.868838501	0.012002245	sarcalumenin-like protein	0	BAG30721.1
BGIBMGA002259	2268	20	-7.943687477	0.012291328	myosin regulatory light chain 2	1.27E-66	NP_001091813.1
BGIBMGA002626	416	9	-6.648925364	0.012291328	antennal binding protein	3.46E-72	NP_001106744.1
BGIBMGA002548	4691	20	-8.992162338	0.013515161	cuticular protein RR-1 motif 4	3.61E-38	NP_001036835.1
BGIBMGA004035	1226	22	-6.918722292	0.017870493	chemosensory protein	1.89E-60	NP_001037066.1
BGIBMGA004894	438	12	-6.308235206	0.020177428	fau-like protein	1.62E-23	XP_002097124.1
BGIBMGA001202	1423	26	-6.892690875	0.020339125	muscle lim protein	0	NP_001103762.1
BGIBMGA000328	2063	29	-7.270957758	0.02122278	cuticular protein RR-1 motif 42	2.08E-75	NP_001166712.1
BGIBMGA014126	374	11	-6.205873488	0.022363186	another b-box isoform c-like protein	7.00E-119	NP_001166752.1
BGIBMGA014227	7874	29	-9.203310649	0.026886154	myosin heavy chain-like protein	0	BAG30740.1
BGIBMGA010111	223	6	-6.334348046	0.027138743	–NA–	-	-
BGIBMGA004103	1816	35	-6.815676117	0.028218303	heat shock protein 1	2.67E-99	NP_001091767.1
BGIBMGA001862	2842	40	-7.269173391	0.030120152	cuticular protein glycine-rich 17	9.00E-40	NP_001166787
BGIBMGA004546	86	0	-Inf	0.030120152	projectin-like protein	0	ADY75709.1
BGIBMGA000336	535	20	-5.859877633	0.031475336	cuticular protein RR-1 motif 34	2.86E-105	NP_001166717.1
BGIBMGA011128	84	0	-Inf	0.032853641	fatty-acyl reductase 2-like protein	8.23E-49	ADI82775.1
BGIBMGA005277	14398	32	-9.93199145	0.034333874	cuticular protein RR-1 motif 2	7.00E-76	NM_001043362
BGIBMGA012763	352	14	-5.770487344	0.034333874	prophenoloxidase subunit 1	0	NP_001037335.1
BGIBMGA013700	2048	43	-6.692145892	0.035777117	troponin T-like protein	1.21E-139	NP_001040221.1
BGIBMGA001201	1911	47	-6.463933858	0.042710356	muscle protein 20	3.32E-103	NP_001040476.1
BGIBMGA008861	442	20	-5.584385112	0.042710356	troponin c 25d	1.12E-80	NP_001040443.1

“Intact" and “Cultured" columns indicate the number of read counts in intact fat body and cultured fat body of *Bombyx mori*.

## Discussion

The transcriptome of lepidopteran fat body was analyzed using next-generation sequencing. Approximately 60% of reads obtained for *Bombyx mori* fat body transcripts were aligned with the genome of silkworm; this result was similar to the result from a previous study on silk glands (52.7% and 60.2%) [19]. Lipoprotein-encoding transcripts occupied approximately half of the transcriptome in intact fat body; in contrast, these transcripts occupied a smaller portion of the transcriptome of fat body tissues that had been maintained in culture medium for 90 h. This result indicated that the cultured tissue had initiated the process of abrogating their tissue-specific function by becoming independent from the donor and their identity as a part of an individual, living organism. The independence of cultured fat body was also indicated by an increase in Shannon’s entropy of RPKMs. This finding indicated that gene expression in the fat body that resulted from inter-tissue communications between the constituent tissues of the insect donor had become dysregulated in the cultured fat body.

Expression of sugar-transporter genes (BGIBMGA004427, BGIBMGA004426) increased during culture of fat body, indicating that, under culture conditions, cells take up sugar actively. Expression of immunity-related genes (BGIBMGA004869, BGIBMGA012864, BGIBMGA013866) also increased during culture, indicating that the process of dissection from donor, the transfer into culture medium, and maintenance in culture medium are harmful for fat body tissue and could stimulate immune responses. In addition, expression of genes related to juvenile hormone synthesis (BGIBMGA002314, BGIBMGA001324) also increased during culture, and expression of cuticle protein genes (BGIBMGA000329 etc) decreased. Expression of genes related to hypoxia (BGIBMGA004893, BGIBMGA004894) also decreased, indicating that culture conditions were oxygen rich. Microsensor measurements of physically dissolved O_2_ in the hemolymph of living buzzer midge larvae indicate very low O_2_ concentrations (11 µmol/L O_2_) [20]; this empirically determined concentration is lower than that in insect culture media [21]. Expression of troponin genes (BGIBMGA001030 etc.) and myosin genes (BGIBMGA014226 etc.) also decreased during culture, indicated that the fat body explants contained some myocytes.

The expression of genes relating to a particular function or processes may have changed in a coordinated fashion during culture. To assess whether such changes occurred, RPKMs were compared for 147 immunity-related genes [22], 54 apoptosis-related genes [23], and 10 juvenile hormone-related genes [24]. Of the 147 immunity-related genes, expression of 60 was increased more than 5-fold during culture; in two extreme examples, expression of the genes encoding gloverins and a gene encoding lebocine increased more than 1000-fold. Expression of most of the 54 apoptosis-related genes did not change during culture. Expression of all 10 genes related to juvenile hormone synthesis increased during culture, and expression 6 of these genes increased more than 5-fold. Cultured tissues were derived from living donor organisms, which have circulating hormones, and were transferred into culture medium lacking hormones. To avoid exposing cultured explants to abrupt changes in insect hormone levels, 5^th^ instar larva were harvested 3 days after the 4^th^ ecdysis; these larva have juvenile hormone and 20-hydroxyecdysone levels that are lower than those of other developmental stages. Hence, cultured fat body would not progress according to the original program of development.

A previous study on a *Drosophila* cell line demonstrated that expression of 5 characteristic genes was elevated in continuously cultured cell lines [25]. However, in the current study, the silkworm homologs of these five genes were not upregulated in the cultured fat body, indicating that increased expression of these genes was not necessary for survival in culture for silkworm cells or tissue explants. These five genes may be essential to maintenance of immortal cells, but not persistence in primary culture conditions. However, further study of these genes in silkworm cell line is warranted.

Culture conditions are very important to cell culture; these conditions comprise the complete environment provided for survival of cultured cells. The cells used in this study were fat body cells from the silkworm, and the medium used was MGM-450 insect medium [26]. The silkworm *B. mori* is one of the few insects for which a complete genome sequence has been compiled, and representative species of lepidopteran insects account for the majority of agricultural pests; therefore, there is substantial interest in gene expression in lepidopteran species. Many studies have specifically focused on fat body because this tissue is largely responsible for resistance to insecticides. The MGM-450 insect medium was originally formulated based on Grace medium which is an improvement of Wyatt’s medium [5]. Wyatt’s medium was formulated as a synthetic culture medium based on the chemical analyses of hemolymph from several insect species including silkworm [27]. MGM-450 insect medium and silkworm hemolymph have similar proportion of amino acids. Material constituting MGM-450 insect medium are 6 inorganic salts, 21 amino acids, 4 other organic acids, 9 vitamins, 3 sugars, polyvinylpyrrolidone, fetal bovine serum, bovine plasma albumin, fetuin, cytochrome c, inosine and tryptose phosphate broth. Six silkworm cell lines were established using MGM-450 insect medium [28], [29] and a research about *in vitro* hemocyte differentiation of silkworm also performed using this medium [30].

**Figure 4:**
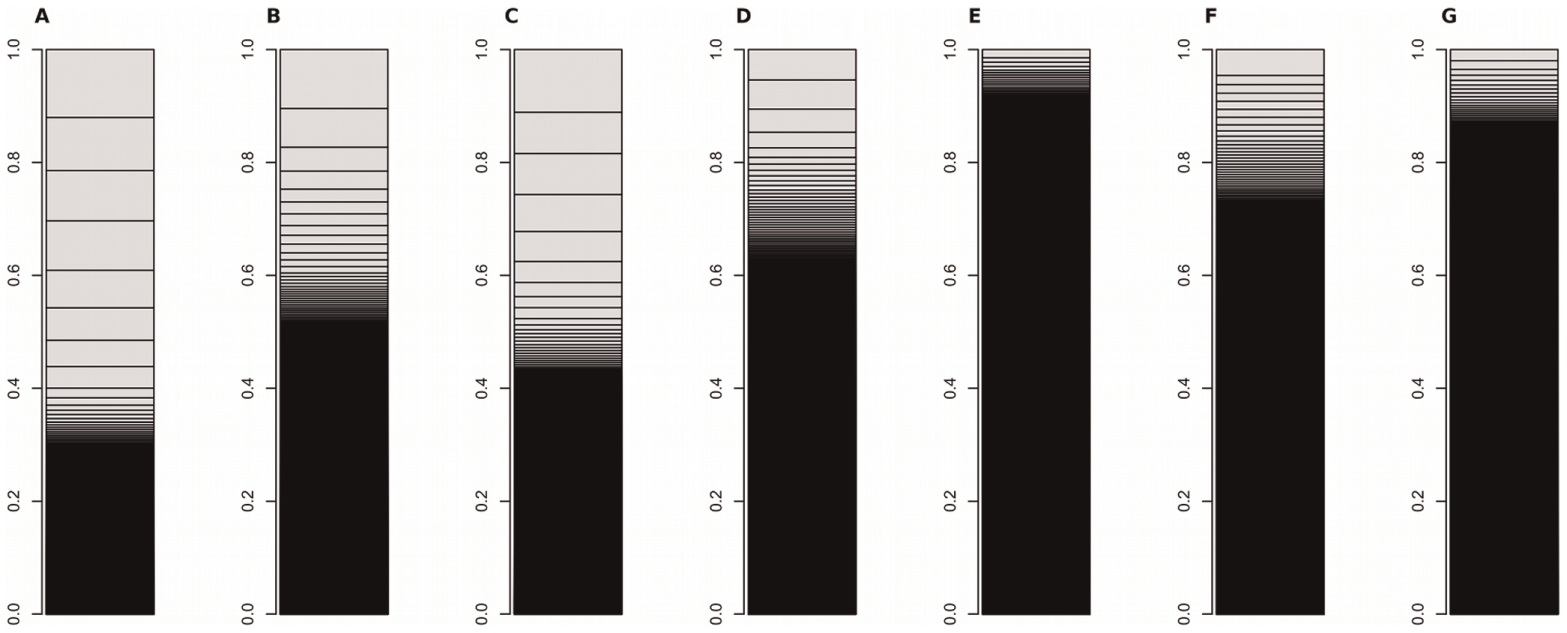
Comparison of transcriptomes using read-count data. A: *Bombyx mori* intact fat body, B: *Bombyx mori* cultured fat body, C: *Drosophila melanogaster* intact fat body, D: *Drosophila melanogaster* intact midgut, E: *Drosophila melanogaster* Kc167 cell line, F: *Homo sapiens* liver, G: *Homo sapiens* kidney.

**Figure 5:**
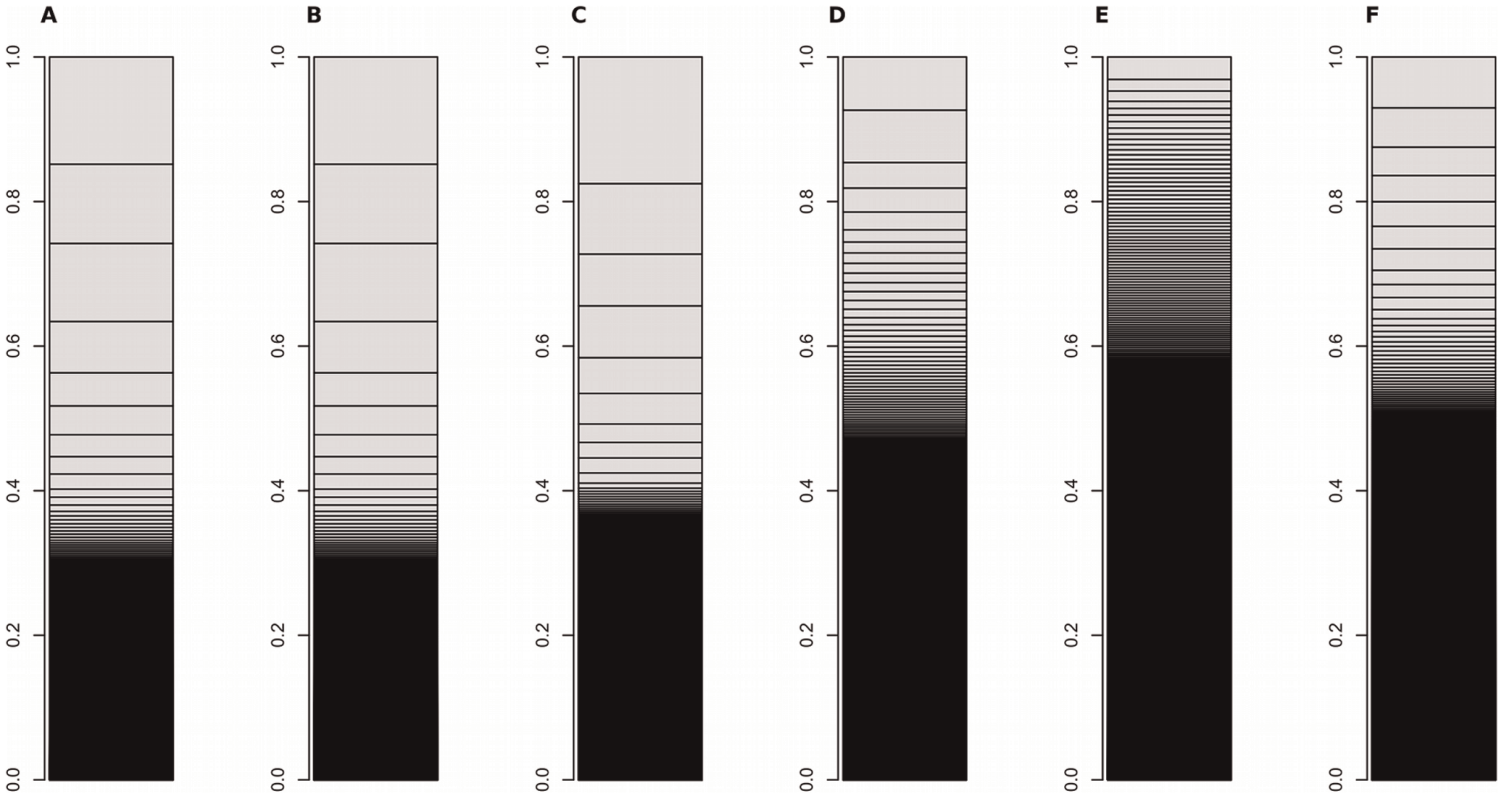
Comparison of transcriptomes using RPKM or FPKM data. A: *Bombyx mori* intact fat body, B: *Bombyx mori* cultured fat body, C: *Drosophila melanogaster* intact fat body, D: *Drosophila melanogaster* intact midgut, E: *Drosophila melanogaster* Kc167 cell line, F: *Mus musculus* muscle.

This study also revealed that fewer genes occupied more of transcriptome in intact fat body than in cultured fat body. To understand characteristics of these differences, we compared the share of transcriptome of tissues from different species and of *Drosophila* cell lines as follows: silkworm fat body (our data), cultured silkworm fat body (our data), *Drosophila* larval fat body [31], *Drosophila* larval midgut [31], a *Drosophila* cell line (SRR029023: modENCODE [32]), human liver [33], human kidney [33], and mouse muscle [11] (Fig. 4 and Fig. 5). The insect fat body had characteristic transcriptomes: fewer genes occupied more of the transcriptome in insect fat body when compared with the transcriptome in the *Drosophila* cell line and with those in the mammalian tissues. These comparisons may support the hypothesis that characteristic transcriptomes of insect tissues contribute to the broad diversity of insects in the process of evolution.

## Materials and Methods

### Establishment of the Primary Culture

The p50 strain of the silkworm, *Bombyx mori*, was reared on the fresh leaves from the mulberry, *Morus bombycis*. 5th instar larvae were harvested 3 days after the 4th ecdysis. The larvae were surface-sterilized by submersion in 70 percent ethyl alcohol, and the fat body were pulled out, care being taken not to injure the digestive system. The fat bodies of 6 larvae were dissected and soaked in 0.75 ml of TRIzol LS (Invitrogen, CA, USA); then, the tissues were kept at −80°C until use. More than 100 chunks of fat body tissue left intact as lobes (approx. 2 mm^3^) were excised from fat bodies dissected from 24 larvae. These tissue particles were incubated in cell culture dishes (ø = 35 mm; BD Biosciences, NJ, USA) with 2ml of MGM-450 insect medium and with no gas change. The tissue particles were cultured without antibiotics for 90 hours at 25°C. We examined the morphology of cultured fat body cells by stereoscopic microscope (Leica EZ4D) and by phase contrast microscope (Leica DM IRB). The presence or absence of microbial infection of the culture was monitored by microscopic inspection. The culture was terminated by soaking the tissues in 0.75 ml of TRIzol LS (Invitrogen, CA, USA), and the tissue particles were kept at −80°C until use.

### RNA Isolation

Total RNA was extracted following the manufacturer’s instructions using TRIzol LS (Invitrogen, CA, USA). Silkworm fat bodies soaked in TRIzol LS were homogenized. The homogenates were incubated for 5 minutes at 25°C. Chloroform (0.2 ml) was added to each sample, the homogenates were shaken vigorously for 15 seconds and then incubated at 25°C for 15 minutes. Samples were subjected to centrifugation at 12,000 × g for 15 minutes at 4°C; subsequently, the aqueous phase of each sample, which contained the RNA, was placed into a new tube; and RNA precipitated out of solution when each aqueous layer was mixed with isopropyl alcohol and these mixtures were subjected to centrifugation at 12,000 × g for 15 minutes at 4°C The RNA pellets were washed twice with 75% ethanol and then dissolved in 40 µl of distilled water. More than 6.9 µg of total RNA was obtained from each sample. The A260/280 ratio was between 1.98 and 2.03 for each RNA sample. The integrity of rRNA in each sample was checked by agarose gel electrophoresis [34].

### Library Preparation and Sequencing: RNA-seq

All libraries were prepared using the mRNA-Seq Sample Preparation Kit (Illumina, San Diego, CA USA) according to the manufacturer’s instruction. In brief, magnetic beads containing poly-T molecules were used to purify mRNA from 10 µg of total RNA. Samples of purified mRNA were then chemically fragmented and reverse transcribed into cDNA. Finally, end repair and A-base tailing was performed before Illumina adapters were ligated to the cDNA fragments. After a gel-size fractionation step to extract fragments of approximately 200 bp, 30 ml of each purified samples was used to amplify cDNA over 15 cycles of PCR. Amplified material was validated and quantified using an Agilent 2100 bioanalyzer and the DNA 1000 Nano Chip Kit (Agilent, Technologies, Santa Clara, CA, USA). Each library was diluted to 10 nM, and 8 pM of each library was loaded onto cBot (Illumina) for cluster generation with cBot Single Read Cluster Generation Kit (Illumina). Sequencing reactions (36 cycles) were performed on a Genome Analyzer IIx (Illumina) using the 36 Cycle Sequencing Kit v4 (Illumina).

### Data Analysis and Programs

Sequence read quality was controlled using FastQC program (http://www.bioinformatics.bbsrc.ac.uk/projects/fastqc/). Short-read sequences were mapped to annotated silkworm genome sequence obtained from KAIKOBASE (http://sgp.dna.affrc.go.jp/) using the CASAVA mapping algorithm. A maximum of two mapping errors were allowed for each alignment. Multi-hit alignments were discarded. Alternatively, at most two internal N characters are permitted. Scaffolds and contigs data were organized with the locus information, which was integrated with a consensus gene set by merging GLEAN on the chromosomes. We defined the adaptor of the mRNA Sequencing Sample Preparation Kit (Illumina), mitochondrial DNA (32 sequences), ribosomal RNA (39 sequences), and transfer RNA (56 sequences) as contaminant sequences. The reads were then aligned by tile onto the reference sequences of *Bombyx mori*. In this study, we focused only on annotated loci. Between 57.89% and 59.56% of the reads were mapped to annotated sequences in the *Bombyx mori* genome, and 11,545 genes out of the 14,624 predicted genes were confirmed to be expressed in the 2 samples. The genome-wide transcript profiles compared between the samples. All the statistical analyses were performed using R software version 2.13.0, the edgeR package, and the DEseq package. The trimmed means of the M values (TMMs) for data normalization were calculated using cNormFactors of edgeR. Normalization of read numbers between samples and differential expression analysis was performed using DEseq. The homology search and local alignments were determined using Blast2go [35]. Sequence data from *Drosophila melanogaster* fat body (SRR073280), *Drosophila melanogaster* midgut (SRR072381), and the *Drosophila melanogaster* Kc167 cell line (SRR029023) were obtained from DDBJ Sequence Read Archive (http://trace.ddbj.nig.ac.jp/dra/index.shtml). Short-read sequences were mapped to the fly genome obtained from Ensembl (http://uswest.ensembl.org/index.html) using Bowtie [36] (options: -n 2 -l 36 –best; http://bowtie-bio.sourceforge.net/index.shtml). To estimate fragments per kilo base of exon per million mapped fragments (FPKM), Tophat [37] (http://tophat.cbcb.umd.edu/) and Cufflinks [38] (http://cufflinks.cbcb.umd.edu/) were used.
